# Privacy, Governance and Public Acceptability in Population Data Linkage for Research

**DOI:** 10.23889/ijpds.v1i1.405

**Published:** 2017-04-19

**Authors:** Christine M O'Keefe

**Affiliations:** 1 CSIRO, Australia

## Introduction

For several years, Population Data Linkage initiatives around the world have been successfully linking population-based administrative and other datasets and making extracts available for research under strong confidentiality protections1For a list of administrative data linkage centres around the world, see www.ipdln.org/data-linkage-centres. This paper provides an overview of current approaches in a range of scenarios, then outlines current relevant trends and potential implications for population data linkage initiatives.

## Methods

Approaches to protecting the confidentiality of data in research can also reduce the statistical usefulness, and the trade-off between confidentiality protection and statistical usefulness is often represented as a Risk-Utility map [2, 3, 5, 7]. Positioning the range of current approaches on such a Risk-Utility map can indicate the relative nature of the trade-off in each case.

Such a Risk-Utility map is only part of the story, however. Each approach needs to be implemented with appropriate levels of governance, information technology security, and ethical oversight. In addition, there are several changes in the external environment that have potential implications for population data linkage initiatives.

## Results and Discussion

Current approaches to protecting the confidentiality of data in research fall into one of two classes. The first class comprises approaches that anonymise the data before analysis, namely:

- Removal of identifying information such as names and addresses
- Secure data centres on-site at the custodian premises
- Public use files made widely available
- Synthetic data files made widely available
- Open data files published on the internet

The second class comprises approaches that anonymise the analysis outputs, namely:

- Virtual data centres that are on-line versions of secure data centres [8]
- Remote analysis centres where users can request analyses but cannot see data..

Many such initiatives implicitly or explicitly use criteria that have been recently captured in the Five Safes model [3]. However, changes in the external environment may add potential implications to address [6].

First, there is a rapid increase in scenarios for data use, many of which involve multiple datasets from multiple sources with multiple custodians. This raises the question of whether there should be centralised data integration versus a proliferation of ad-hoc decentralised but inter-related initiatives. In any case, harmonised and shared governance will be essential. Next, the public are becoming increasingly informed and are increasingly exercising their privacy preferences in selecting between competing service providers. It is likely that the public will demand that initiatives move beyond education gain acceptance to a model of full partnership.

## Conclusions

While Population Data Linkage initiatives have been successful to date, changes in the external environment have potential implications such as a need for harmonised and shared governance, as well as full partnership with the public. Meeting the future challenges will require sophistication in the selection, design and operation of approaches to protecting the confidentiality of data in research. Useful frameworks in this context include [1, 4]. Importantly, it is necessary to have a range of approaches in order to adequately meet the needs of a range of different scenarios.

**Figure 1:**
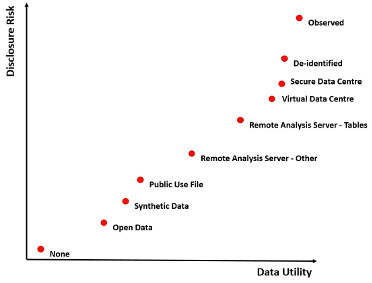
Risk-Utility map indicating the nature of the trade-off between confidentiality protection and statistical usefulness for a range of popular current approaches
